# ADAMTS13: An Emerging Target in Stroke Therapy

**DOI:** 10.3389/fneur.2019.00772

**Published:** 2019-07-17

**Authors:** Xin Chen, Xin Cheng, Shufan Zhang, Danhong Wu

**Affiliations:** ^1^Department of Neurology, Shanghai Fifth People's Hospital, Fudan University, Shanghai, China; ^2^Department of Neurology, Huashan Hospital, Fudan University, Shanghai, China

**Keywords:** ADAMTS13, VWF, structure, physiological function, ischemic stroke

## Abstract

Thrombosis is the predominant underlying mechanism of acute ischemic stroke (AIS). Though thrombolysis with tPA has been proven to be effective in treating AIS within the time window, the majority of AIS patients fail to receive tPA due to various reasons. Current medical therapies for AIS have limited efficacy and pose a risk of intracerebral hemorrhage. ADAMTS13 (a disintegrin and metalloprotease with a thrombospondin type 1 motif, member 13) is a metalloprotease that effectively breaks down the von Willebrand Factor (VWF), a key factor in thrombus formation. Previous studies have proven that dysfunction of ADAMTS13 is associated with many diseases. Recently, ADAMTS13 has been reported to be closely related to stroke. In this review, we briefly described the structure of ADAMTS13 and its role in thrombosis, inflammation, as well as angiogenesis. We then focused on the relationship between ADAMTS13 and AIS, ranging from ischemic stroke occurrence, to AIS treatment and prognosis. Based on research findings from *in vitro*, animal, and clinical studies, we propose that ADAMTS13 is a potential biomarker to guide appropriate treatment for ischemic stroke and a promising therapeutic agent for tPA resistant thrombi.

## Introduction

Stroke is one of the leading causes of death and disability in both developing and developed countries (1). Ischemic stroke accounts for approximately 85–90% of all types of stroke (2). Currently, intravenous thrombolysis with tissue plasminogen activator (t-PA) is the most efficacious treatment for AIS patients when applied within 4.5 h after the onset of symptoms (3). However, over 90% of ischemic stroke patients did not receive tPA due to the short time window and the increased risk of intracerebral hemorrhage when tPA is applied out of this time window (4). A similar situation is also observed in secondary prevention of stroke. For example, antiplatelets and anticoagulants show limited efficacy in reducing recurrent strokes, but they significantly increase the risk of intracerebral hemorrhage (5, 6). Therefore, there is a demanding need to further explore the underlying mechanisms of AIS in order to develop novel treatments.

ADAMTS13 (a disintegrin and metalloprotease with thrombospondin type 1 repeats 13), also known as the von Willebrand factor-cleaving protease (VWFCP), is predominantly synthesized in the liver. It cleaves the ultra-large molecule—von Willebrand factor (VWF), a key player in initiating platelet tethering and subsequent platelet adhesion (7), into smaller and less reactive molecules (8). It is well-known that dysfunction of ADAMTS13 is associated with diverse diseases, such as thrombotic thrombocytopenic purpura, pre-eclampsia, acute myocardial infarction, and diabetes. Recently, the relationship between ADAMTS13 and ischemic stroke has become a focus of stroke research. Both animal and clinical studies have demonstrated the important role ADAMTS13 plays in the pathogenesis of ischemic stroke, suggesting that ADAMTS13 might be a promising therapeutic target for ischemic stroke.

### ADAMTS13: Structural Features

ADAMTS13, originally named VWFCP, was purified from the human plasma for the first time in 1996 (9). Its gene was identified in 2001 and it was renamed ADAMTS13 based on its partial amino acid sequence (10). Thereafter, information about the structure and function of ADAMTS13 has been revealed. The delicate relationship between ADAMTS13 and thrombosis also becomes an intriguing subject.

**Figure 1:**
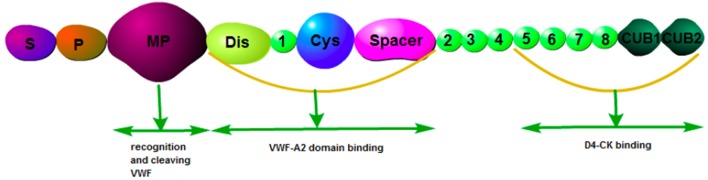
Structure of ADAMTS13. S, signal peptide domain; P, short propeptide domain; MP, metalloprotease domain; Dis, disintegrin-like domain; 1, thrombospondin-1 repeat (TSP1) domain; Cys, characteristic Cysteine -rich domain; Spacer, spacer domain; CUB, CUB domains.

**Figure 2:**
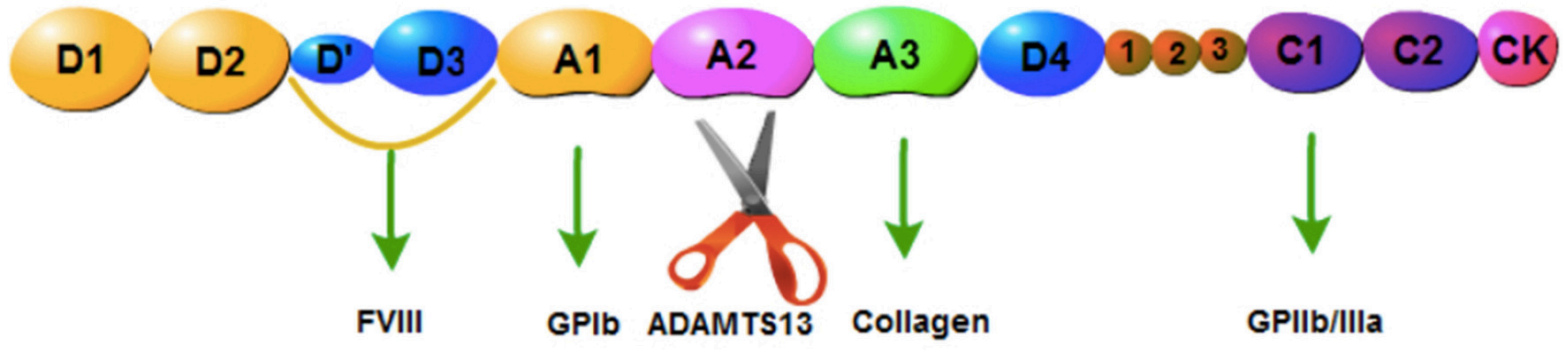
Structure of VWF. D1,D2: pro-VWF, D'D3: FVIII, A1: GPIbα protein addition site, A2: ADAMTS13 cleaving site, A3: collagen.

ADAMTS13, a metalloprotease containing 1,427 amino acid residues, is predominantly secreted by hepatic stellate cells (11). It has been reported that endothelial cells also express ADAMTS13 mRNA and protein (12). The physiological function of ADAMTS13 is based on its multi-domain structure consisting of a signal peptide domain, a short propeptide domain, a metalloprotease domain, a disintegrin-like domain, a thrombospondin-1 repeat (TSP1) domain, a characteristic Cys-rich domain, a spacer domain, and two CUB domains (10) (Figure 1). ADAMTS13 is the cleaving protease of VWF, which is a large multimeric glycoprotein. VWF is released by endothelial cells in the form of ultra-large multimers (UL-VWF) of varying sizes, with the molecular weight up to 20,000 kDa. ADAMTS13 cleaves the Y1605-M1606 bond within the UL-VWF A2 domain (Figure 2). Dysfunction of the ADAMTS13-VWF axis leads to VWF accumulation and adhesion of platelets, which is the first step of thrombosis and inflammation (13, 14).

Recently, more functions of ADAMTS13 have been revealed with the identification of a substrate-induced conformational activation mechanism. ADAMTS13 circulates in a quiescent form, maintained by an autoinhibitory interaction between its N-terminal spacer domain and its C-terminal CUB-domains (15). Its activity is significantly increased upon binding to the D4-CK domain of the globular VWF exposing the spacer domain exosite, enabling efficient proteolysis of VWF. Exposure of ADAMTS-13 to the VWF D4-CK domain results in approximately a 2.5-fold enhancement of its activity (15). Furthermore, it has been reported in an ADAMTS13 activation model that the VWF D4-CK domain engages the TSP8-CUB2 domains, disrupting the CUB1-spacer domain interaction and consequently activating ADAMTS13 (16). A recent stability and dynamics study on ADAMTS13 conformation showed that wild type ADAMTS13 adopted two distinct conformational states (state I and state II) at pH 6 and pH 7.5, respectively, whereas a gain-of-function variant ADAMTS13 showed an additional state (state III). They proposed that low pH might alter the tertiary structure and/or disrupt the intra-domain interaction, and as a result, the flexibility of ADAMTS13 was increased (17).

### ADAMTS13: Roles in Thrombosis, Inflammation and Angiogenesis

The critical role of ADAMTS13 in thrombosis is exemplified by its importance in the pathogenesis of thrombotic thrombocytopenic purpura (TTP). TTP is a life-threatening microangiopathic disorder of the blood-coagulation system, caused by the deficiency of ADAMTS13 either in quantity or in quality (18). Mutations in the ADAMTS13 gene are one of the key pathological mechanisms of congenital TTP, whereas, heterogeneous and polyclonal autoimmune response against ADAMTS13 is the critical mechanism for acquired TTP (8).

Adhesion of platelets is the first step not only in thrombosis but also in inflammation mediated by VWF. ADAMTS13 down-regulates both thrombosis and inflammation through cleavage of VWF. Previous studies have demonstrated that ADAMTS13 prevents the formation of thrombi in injured microvenules and regulates thrombosis in arterioles by altering the interaction between platelets and VWF (19, 20).

It is well-known that rolling and adhesion of leukocytes at the site of infection or injury are one of the main features of inflammation, which is influenced by a variety of factors (21–24). A study reported that VWF could promote both leukocyte rolling and stable adhesion by providing binding sites for two leukocyte receptors—P-selectin glycoprotein ligand 1 and beta 2 integrin (25). It also facilitates neutrophil extravasation from blood vessels (26). A number of molecules can influence the activity of VWF, such as platelet glycoprotein Ibα (GPIbα), N-Acetylcysteine, and plasmin. The interaction between GPIbα and VWF not only initiates hemostasis after vascular injury, but also contributes to pathological thrombosis (27). N-acetylcysteine reduces the size and activity of VWF in both the human and the mouse plasma (28). An *in vitro* experiment has shown that plasmin cleaves VWF at K1491-R1492 in the A1-A2 linker region in a shear- and glycan-dependent manner (29). Therefore, ADAMTS13 plays a pivotal role in thrombosis and inflammation through cleaving VWF. Animal and human studies have confirmed the essential roles that ADAMTS13 plays. Deficiency in ADAMTS13 results in increased leukocyte rolling and adhesion in both unstimulated and inflamed veins (30, 31). Chemotherapies that decrease vascular endothelial growth factor (VEGF) have been shown to result in thrombotic microangiopathies (TMAs, TTP is the major type of TMAs) (32). Deficiency in ADAMTS13 endopeptidase contributes to the development of VEGF inhibitor-related thrombotic microangiopathies (33). Patients with sepsis or DIC have decreased levels of ADAMTS13 and increased levels of VWF (34). In mice, deficiency in ADAMTS13 (ADAMTS13^−/−^) leads to aggravated inflammatory and thrombotic responses. Treatment with ADAMTS13 decreases the severity of colitis through its anti-inflammatory mechanism (35). Similar results were observed in a mouse study where rhADAMTS13 was shown to protect kidneys against the ischemia/reperfusion injury by attenuating apoptosis as well as inflammation, and rectifying endothelial dysfunction (36). However, patients with severe ADAMTS13 deficiency do not always develop TTP. There must be other factors that influence the pathogenesis of this disease. Apart from infection, the most common trigger (41% of episodes) of TTP, excessive alcohol consumption, pregnancy, drug/medication use, injury, food poisoning, and various others could also trigger TTP in individuals carrying homozygous or heterozygous mutations in the ADAMTS13 gene (37, 38). Similar results were also found in animal studies. Motto et al suggested that microbe-derived toxins (or possibly other sources of endothelial injury), together with genetic susceptibility, were required to trigger TTP in settings of ADAMTS13 deficiency (39). Severe ADAMTS13 deficiency in mice was not sufficient to cause TTP-like symptoms (40). Therefore, complete deficiency in ADAMTS13 seems to result in a prothrombotic state, which is an important risk factor for TTP or stroke, but it is insufficient to cause TTP or stroke by itself. Additional genetic or environmental factors are needed to trigger TTP or stroke.

It has been reported that the active metalloprotease domain of other members of the ADAMTS family increases the production of growth factors and cytokines by degrading the extracellular matrix components. Subsequently, they can accelerate cell proliferation and angiogenesis (41, 42). Whereas, TSP1 has been proven to suppress angiogenesis by regulating the activity of VEGF (43). Based on the similarity of the structure of ADAMTS13 to other members of the ADAMTS family, which contain the metalloprotease domain and the TSP-1 domain, ADAMTS13 is very likely to be involved in angiogenesis. This is confirmed by an *in vitro* study where the full-length ADAMTS13 promotes angiogenesis when it is incubated with human umbilical vein endothelial cells (HUVEC), but it counteracts the angiogenic activity of VEGF (44). A later study demonstrated that angiogenesis induced by ADAMTS13 or its variants was through upregulating the production of VEGF and phosphorylation of VEGFR2, and the C-terminal TSP1 repeats of ADAMTS13 dominated the angiogenic activity (45).

### ADAMTS13 and Stroke

Due to the close relationship between VWF and platelet aggregation as well as thrombus formation, research has focused on the correlation between high levels of VWF and cardiovascular/cerebrovascular diseases including ischemic stroke. Prospective studies have reported that high levels of VWF increase the risk of developing AIS (46, 47). A study even showed the correlation between levels of VWF and subtypes of stroke as well as functional outcomes in AIS patients (48). It was proposed that the imbalance between VWF and ADAMTS13, a down-regulator of VWF, might be the underlying mechanism of growing thrombus (46, 49). Complete lack of ADAMTS13 in mice does not necessarily result in stroke, but does lead to a prothrombotic state (40). Furthermore, ADAMTS13 mediated angiogenesis plays an important role in the pathogenesis of ischemic stroke (44, 45). It is, therefore, logical to anticipate a strong correlation between low levels of ADAMTS13 and the development of ischemic stroke.

### ADAMTS13 and Stroke Occurrence

Currently, there is no conclusion on the relationship between ADAMTS13 and the outcome of ischemic stroke. A study reported that patients with cardiovascular diseases had a lower level of ADAMTS13 than healthy controls, whereas patients with ischemic stroke had a similar level of ADAMTS13 to healthy controls (50). Another study showed that levels of ADAMTS13 in AIS patients were significantly lower than those in normal subjects as well as in chronic cardiovascular disease patients (51). In addition, a number of studies reported a significant association between low activity/ levels of ADAMTS13 and stroke (46, 52–54). It was discovered that ADAMTS13 single nucleotide variants were closely associated with pediatric stroke susceptibility (55). The difference between these research results may be explained by differences in the study inclusion criteria because infection, excessive alcohol consumption, pregnancy, drug/medication use, injury, food poisoning, and others can influence the impact of ADAMTS13 on stroke (37, 38). In a prospective cohort study, the activity of plasma ADAMTS13 was tested in 5,941 volunteers who were over 55 years old without a history of stroke or transient ischemic attack. It was found that individuals with lower ADAMTS13 activities had a higher risk of ischemic stroke than those with normal ADAMTS13 activities (52). Importantly, correlation between ADAMTS13 activities and the incidence of stroke persisted after adjusting for common risk factors such as age, sex, diabetes and atrial fibrillation. These indicate that low levels of ADAMTS13 are an independent predictor of stroke. Similar results were also found in pediatric ischemic stroke studies (56, 57). It was proposed that a decrease in activities or levels of ADAMTS13 would reduce the cleavage of ULVWF, resulting in the formation of larger VWF polymers and a hypercoagulative state at the site of vascular injury. Consequently, thrombosis may occur (52). Recently, the CHA2DS2-VASc Score (congestive heart failure, hypertension, age≥75 years [doubled], diabetes mellitus, stroke/transient ischemic attack/thromboembolism [doubled], vascular disease [prior myocardial infarction, peripheral artery disease, or aortic plaque], age 65–74 years, and sex category [female]) is recognized as a useful tool to assess the risk of developing ischemic stroke due to multiple risk factors. In a prospective study, 196 old patients (≥60 years) with and without non-valve atrial fibrillation (AF) were recruited and their baseline information was compared. It was found that there was a significant correlation between plasma levels of VWF, ADMATS13, and the CHA2DS2-VASc Score in older patients with and without AF (58). At the molecular level, ADAMTS13, one of the extracellular matrix components, in conjunction with phosphoinositide and calcium signaling pathways, plays a vital role in determining the susceptibility for stroke (57). Although there is no conclusion on the relationship between ADAMTS13 and stroke severity, Denorme et al found that the activity of ADAMTS13 in patients with ischemic stroke was similar to that in patients with transient ischemic attack. They speculated that the activity of ADAMTS13 can not predict the severity of ischemic stroke. However, they found that the VWF:ADAMTS13 ratio was significantly associated with stroke severity and modality (51). No significant difference was found in the activity of ADAMTS13 between different subtypes of stroke (steno-occlusive arteriopathies, cardioembolic, undetermined) (56).

Taken together, it is clear that levels of ADAMTS13, as well as levels of VWF, are associated with the risk of stroke in the general population. The VWF: ADAMTS13 ratio has a stronger correlation with the risk of stroke than either of them. However, larger prospective studies and standardized test systems are further needed before determining whether levels of VWF and ADAMTS13 can be used as a clinical marker to predict the risk of cardiovascular/cerebrovascular diseases in individual patients.

### Experimental Studies: Role of ADAMTS13 in Acute Ischemic Stroke

The importance of decreased ADAMTS13 as an important risk factor for ischemic stroke in humans has promoted experimental trials in acute stroke models. It was reported that the volume of the cerebral infarct was significantly reduced with decreased VWF levels after ischemic stroke, whereas the volume of the cerebral infarct was increased with decreased ADAMTS13 levels. Infusion of ADAMTS13 before reperfusion significantly reduced the volume of infarcts and improved functional outcomes (59). A study on ischemia-reperfusion injury reported that the regional blood flow of the ischemic cortex was notably decreased in ADAMTS13^−/−^mice after reperfusion compared with wild-type mice (60). Since thrombus formation after reperfusion is related to the accumulation of platelets, the authors proposed that ADAMTS13 protects the brain from ischemic injury through mediating VWF-platelet interactions (60). Apart from decreased blood flow, accumulation of inflammatory cells was observed in the brains of ADAMTS13^−/−^mice, suggesting that ADAMTS13 may protect the brain from ischemia by regulating VWF-dependent inflammation after reperfusion (60). Similar results were found in another study which demonstrated increased levels of inflammatory cytokines and neutrophil infiltration in ADAMTS13^−/−^mice after ischemic stroke. Opposite results were found in VWF^−/−^mice (61). It is concluded that ADAMTS13 protects the brain tissue from ischemic injury by regulating VWF-mediated inflammation and thrombosis. As previous studies have shown that complete deficiency in ADAMTS13 alone was not sufficient to cause TTP, additional genetic or environmental factors were required (39, 40). This also applies to animal studies.

There are types of VWF and they have different roles in physiological and pathological conditions. Dhanesha et al generated platelet-derived VWF (Plt-VWF) mice, Plt-VWF mice with ADAMTS13 deficiency, and endothelial cell-derived VWF (EC-VWF/ADAMTS13^−/−^) mice through bone marrow transplantation to determine the function of different types of VWF in stroke. They found that no difference was seen in infarct size and post-ischemic inflammation between Plt-VWF and Plt-VWF/ADAMTS13^−/−^ mice, but the infarct size and post ischemic inflammation were significantly aggravated in EC-VWF and wild-type mice and attenuated in VWF^−/−^ mice compared with those of Plt-VWF or Plt-VWF/ADAMTS13^−/−^ mice (62). These indicate that ADAMTS13 protects the brain from ischemia-reperfusion injury predominantly through cleaving the endothelial cell-derived VWF. To further clarify the molecular mechanism of the protective effect of ADAMTS13 on stroke, Zhao et al tested the relationship between expression of miR-525-5p and ADAMTS13 and confirmed that the level of miR-525-5p in the brain is significantly decreased after oxygen-glucose deprivation (OGD). Intracerebroventricular injections of an miR-525-5p antagomir into the ischemic stroke rats significantly reduced the volume of cerebral infarcts. Cell culture experiments showed that when cells were transfected with an miR-525-5p agomir, levels of ADAMTS13 mRNA and protein were significantly reduced. Meanwhile, miR-525-5p was found to bind to the 3 '-UTR of ADAMTS13. It has been concluded that miR-525-5p might negatively regulate the expression of ADAMTS13 after stroke. Downregulating the expression of miR-525-5p and consequently increasing the expression of ADAMTS13 might minimize ischemic injury (63).

Though ADAMTS13 does not have subtypes, it does have different conformations. It has been reported that the previously identified, dose-dependent activity of wild-type ADAMTS13 in murine models of stroke could be further improved by conformational preactivation (64). This raises the possibility of developing strategies to increase the activity of ADAMTS13.

### Promising Use of ADAMTS13 in Stroke Therapy

Previous studies have shown that ADAMTS13 may become a promising pharmacological agent for ischemic stroke treatment through regulating VWF-mediated inflammation and thrombus formation in addition to its role in angiogenesis, the latter being especially important for ischemic stroke recovery. Angiogenesis can be initiated by VEGF and VEGF receptor 2 (VEGFR-2), and vascular maturation is also reliant on angiopoietins (Angs) 1 and 2 (65). It was confirmed that VEGF-A and Ang-2 had synergistic effects on angiogenesis (66). Deficiency in VWF in endothelial cells resulted in increased activation of VEGFR-2 and proliferation of endothelial cells as well as increased release of Ang-2 (67). Other VWF-associated angiogenic regulators may be involved in angiogenesis, such as galectin-3 (Gal-3) which could directly bind to VWF and initiate the phosphorylation of VEGFR-2 (68, 69). Therefore, ADAMTS13 is very likely to become a therapeutic agent for ischemic stroke. It has been reported that the activity of ADAMTS13 is reduced in acute myocardial infarction (70). Its activity has also been found to be reduced in the early phase of ischemic stroke (53, 71). Though the relationship between levels of the ADAMTS13 antigen and its activity remains controversial (this may be due to the fact that the activity of ADAMTS13 varies in different pathologic conditions, and it might be influenced by storage conditions) (72), a study found that the reduced level of ADAMTS13 could predict poor response to recanalization therapies in AIS (73). Therefore, ADAMTS13 might be a valuable biomarker to guide reperfusion therapies.

Currently, thrombolysis with tissue plasminogen activator (tPA) remains the most effective therapy for patients with AIS, but it has a high risk of intracerebral hemorrhage. Although studies on the efficacy of ADAMTS13 in managing ischemic stroke is still at the stage of animal research, it has shown advantages and is expected to provide a new direction for the treatment of ischemic stroke. It has been reported that recombinant ADAMTS13 (rADAMTS13), when injected into mice before ischemia reperfusion, significantly reduced the infarct volume and improved prognosis without eliciting intracerebral hemorrhage. To further explore the effect of ADAMTS13 on ischemic stroke, the team injected tPA, VWF, and rADAMTS13 into ischemic stroke mice and tested the permeability of the blood brain barrier along with intracerebral hemorrhage. It was found that 540 ng VWF increased the permeability of the blood brain barrier, which was completely reversed by rADAMTS13. Interestingly, rADAMTS13 alone had no effect on the permeability of the blood-brain barrier. Injections of tPA led to intracerebral hemorrhage and the volume of the hematoma was reduced by rADAMTS13. Injections of rADAMTS13 alone did not lead to intracerebral hemorrhage. Simultaneous injections of both VWF and tPA did not show significant differences in intracerebral hemorrhage from injections of tPA alone (59). A study reported that ADAMTS13 reduced the expression of MMP-9 and an inflammatory factor intercellular cell adhesion molecule-1 (ICAM-1), suggesting that rADAMTS13 protects the brain predominantly by reducing the inflammatory response (53). Similar results were reported by another study (74). Therefore, the combination of rADAMTS 13 and tPA might become a novel treatment for ischemic stroke by diminishing the neurotoxic effect of exogenous tPA. This has shown its potential in an animal study where VWF-rich thrombi in the middle cerebral artery (MCA) of mice were not dissolved by tPA but by ADAMTS13 in a dose-dependent manner. This phenomenon was observed even when ADAMTS13 was administered 60 min after occlusion (75). A Japanese study further explored the effect of tPA and rADAMTS13 on ischemic stroke. ADAMTS13 (0.1 mg/kg) and tPA (1 mg/kg) were injected to mice 2 and 4 h after MCAO, respectively. It was found that rADAMTS13 reduced the infarct volume in both 2 and 4 h MCAO mice without incurring intracerebral hemorrhage. Though tPA could reduce the infarct volume, it led to intracerebral hemorrhage in 4 h MCAO mice. Therefore, rADAMTS13 has a longer time window than tPA for the treatment of AIS, which lays the theoretical foundation for translational research on ADAMTS13 (76).

### ADAMTS13 and Stroke Outcomes

There were few animal studies on the relationship between levels of ADAMTS13 and the prognosis of ischemic stroke, though it is widely believed that there is a significant correlation between them. A recent study showed that cerebral vascular angiogenesis, new muscle cell coverage, and expression of connexin in ADAMTS13^−/−^ mice were significantly lower than those in wild-type mice 14 days after the ischemic stroke onset, and that deposition of destructed blood-brain barrier and peripheral serum proteins were significantly increased in ADAMTS13^−/−^ mice. In contrast, VWF^−/−^ mice showed significantly more new microvasculature after reperfusion than wild type mice. Injections of rADAMTS13 to wild type mice 7 days after stroke onset increased the formation of neovasculature and repair of blood vessels, and significantly improved the 14-day prognosis after stroke (77). This indicates that rADAMTS13 plays a key role in functional recovery after an ischemic vascular injury. rADAMTS13 is expected to become a new drug to improve the prognosis of ischemic stroke.

Due to the close relationship between levels of ADAMTS13 and the occurrence of ischemic stroke, more and more clinical studies have explored the relationship between levels of ADAMTS13 and the prognosis of ischemic stroke. A prospective case-control study in the UK showed that levels of ADAMTS13 in ischemic stroke or TIA patients who received aspirin were reduced in the early stage (≤4 weeks), but increased to normal later (≥3 months). It was speculated that ADAMTS13 might play an important role in counteracting thrombus formation in ischemic stroke. In addition, no correlation was found between levels of ADAMTS13 and subtypes of stroke. This study did not examine the relationship between levels of ADAMTS13 and the prognosis of ischemic stroke patients (71). The Rotterdam study recruited 6,130 individuals who were over 55 years old and tested the activity of ADAMTS13 and levels of VWF. During the follow-up period (11.3 years in average), 1,868 individuals died (30.5%), among whom 442 were due to cardiogenic death (23.7%). The risk of cardiogenic death was high among those with low ADAMTS13 activity or high levels of VWF, and the risk ratio was 1.46 and 1.29, respectively. The risk of cardiogenic death was higher in those with low ADAMTS13 activity and high levels of VWF and the risk ratio was 1.73. These suggest that ADAMTS13 activity and levels of VWF were closely related to the risk of death, especially cardiogenic death (49). In another study, it was found that high levels of blood ADAMTS13 collected before commencing the mechanical endovascular procedure and intra-arterial administration of heparin were independently related to poor clinical outcomes 3 months after mechanical thrombectomy (mRS = 3–6) (78).

## Conclusion

In summary, the structure and function of ADAMTS13 have been elucidated in the past 20 years. It has been proven that ADAMTS13 is closely related to the occurrence, development, and prognosis of ischemic stroke, protecting the brain from ischemia-reperfusion injury. The VWF:ADAMTS13 ratio has a strong correlation with the risk of stroke. Complete deficiency in ADAMTS13 alone does not lead to ischemic stroke but a prothrombotic state, and the latter may result in ischemic stroke in conjunction with additional genetic or environmental factors. The activity and levels of ADAMTS13 have a good predictive value for the occurrence and prognosis of ischemic stroke. In addition, animal studies on ADAMTS13 in the treatment of AIS have made remarkable progress, especially in the model of conformational activation. ADAMTS13 is expected to become a new therapeutic agent for ischemic stroke.

## Author Contributions

XChen, XCheng, and DW devised the review. XChen and XCheng conducted literature review and provided the first draft. XChen, XCheng, and SZ created the figures. All authors contributed to manuscript revision and approved the submitted version.

### Conflict of Interest Statement

The authors declare that the research was conducted in the absence of any commercial or financial relationships that could be construed as a potential conflict of interest.

## References

[1] Feigin VL Nguyen G Cercy K Johnson CO Alam T. Global, Regional, and Country-Specific Lifetime Risks of Stroke, 1990 and 2016. N Engl J Med. (2018) 379:2429–37. 10.1056/NEJMoa1804492 30575491 PMC6247346

[2] Chaturvedi M Kaczmarek L. Mmp-9 inhibition: a therapeutic strategy in ischemic stroke. Mol Neurobiol. (2014) 49:563–73. 10.1007/s12035-013-8538-z 24026771 PMC3918117

[3] Powers WJ Rabinstein AA Ackerson T Adeoye OM Bambakidis NC Becker K. 2018 Guidelines for the early management of patients with acute ischemic stroke: A Guideline for Healthcare Professionals From the American Heart Association/American Stroke Association. Stroke. (2018) 49:e46–110. 10.1161/STR.0000000000000172 29367334

[4] Vishnu VY Padma Srivastava MV. Innovations in Acute Stroke Reperfusion Strategies. Ann Ind Acad Neurol. (2019) 22:6–12. 10.4103/aian.AIAN_263_18 30692752 PMC6327700

[5] Kernan WN Ovbiagele B Black HR Bravata DM Chimowitz MI Ezekowitz MD. Guidelines for the prevention of stroke in patients with stroke and transient ischemic attack: a guideline for healthcare professionals from the American Heart Association/American Stroke Association. Stroke. (2014) 45:2160–236. 10.1161/STROKEAHA.115.008661 24788967

[6] Rothwell PM Algra A Amarenco P. Medical treatment in acute and long-term secondary prevention after transient ischaemic attack and ischaemic stroke. Lancet. (2011) 377:1681–92. 10.1016/S0140-6736(11)60516-3 21571151

[7] Crawley JT de Groot R Xiang Y Luken BM Lane DA. Unraveling the scissile bond: how ADAMTS13 recognizes and cleaves von Willebrand factor. Blood. (2011) 118:3212–21. 10.1182/blood-2011-02-306597 21715306 PMC3179391

[8] Levy GG Nichols WC Lian EC Foroud T McClintick JN McGee BM. Mutations in a member of the ADAMTS gene family cause thrombotic thrombocytopenic purpura. Nature. (2001) 413:488–94. 10.1038/35097008 11586351

[9] Furlan M Robles R Lämmle B. Partial purification and characterization of a protease from human plasma cleaving von Willebrand factor to fragments produced by *in vivo* proteolysis. Blood. (1996) 87:4223–34. 8639781

[10] Zheng X Chung D Takayama TK Majerus EM Sadler JE Fujikawa K. Structure of von Willebrand factor-cleaving protease (ADAMTS13), a metalloprotease involved in thrombotic thrombocytopenic purpura. J Biol Chem. (2001) 276:41059–63. 10.1074/jbc.C100515200 11557746

[11] Haegele S Fuxsteiner J Pereyra D Koeditz C Rumpf B Schuetz C. Elevated ADAMTS13 activity is associated with poor postoperative outcome in patients undergoing liver resection. Sci Rep. (2018) 8:16823. 10.1038/s41598-018-34794-w 30429491 PMC6235878

[12] Wang A Duan Q Wu J Liu X Sun Z. The expression of ADAMTS13 in human microvascular endothelial cells. Blood Coagul Fibrinolysis. (2016) 27:464–6. 10.1097/MBC.0000000000000405 26366828

[13] Witsch T Martinod K Sorvillo N Portier I De Meyer SF Wagner DD. Recombinant human ADAMTS13 treatment improves myocardial remodeling and functionality after pressure overload injury in mice. J Am Heart Assoc. (2018) 7:7004. 10.1161/JAHA.117.007004 29367415 PMC5850234

[14] Koupenova M Clancy L Corkrey HA Freedman JE. Circulating platelets as mediators of immunity, inflammation, and thrombosis. Circ Res. (2018) 122:337–51. 10.1161/CIRCRESAHA.117.310795 29348254 PMC5777300

[15] South K Luken BM Crawley JT Phillips R Thomas M Collins RF. Conformational activation of ADAMTS13. Proc Natl Acad Sci USA. (2014) 111:18578–83. 10.1073/pnas.1411979112 25512499 PMC4284544

[16] South K Freitas MO Lane DA. A model for the conformational activation of the structurally quiescent metalloprotease ADAMTS13 by von Willebrand factor. J Biol Chem. (2018) 293:1149–50. 10.1074/jbc.AAC117.001638 29374083 PMC5787794

[17] Yu S Liu W Fang J Shi X Wu J Fang Y. AFM imaging reveals multiple conformational states of ADAMTS13. J Biol Eng. (2019) 13:9. 10.1186/s13036-018-0102-y 30679946 PMC6343300

[18] Salles II Crawley JT. Blocking VWF platelet binding to treat TTP. Blood. (2012) 120:3390–2. 10.1182/blood-2012-08-448530 23100301

[19] Pillai VG Bao J Zander CB McDaniel JK Chetty PS Seeholzer SH. Human neutrophil peptides inhibit cleavage of von Willebrand factor by ADAMTS13: a potential link of inflammation to TTP. Blood. (2016) 128:110–9. 10.1182/blood-2015-12-688747 27207796 PMC4937355

[20] Gogia S Kelkar A Zhang C Dayananda KM Neelamegham S. Role of calcium in regulating the intra- and extracellular cleavage of von Willebrand factor by the protease ADAMTS13. Blood Adv. (2017) 1:2063–74. 10.1182/bloodadvances.2017009027 29296853 PMC5728285

[21] McEver RP. Selectins: initiators of leucocyte adhesion and signalling at the vascular wall. Cardiovasc Res. (2015) 107:331–9. 10.1093/cvr/cvv154 25994174 PMC4592324

[22] Rabinovich A Cohen JM Kahn SR. Predictive value of markers of inflammation in the postthrombotic syndrome: a systematic review: inflammatory biomarkers and PTS. Thrombosis Res. (2015) 136:289–97. 10.1016/j.thromres.2015.06.024 26139086

[23] Thomas MR Storey RF. The role of platelets in inflammation. Thrombosis Haemostasis. (2015) 114:449–58. 10.1160/TH14-12-1067 26293514

[24] Fan Z Ley K. Leukocyte arrest: Biomechanics and molecular mechanisms of beta2 integrin activation. Biorheology. (2015) 52:353–77. 10.3233/BIR-15085 26684674 PMC4869873

[25] Pendu R Terraube V Christophe OD Gahmberg CG de Groot PG Lenting PJ. P-selectin glycoprotein ligand 1 and beta2–integrins cooperate in the adhesion of leukocytes to von Willebrand factor. Blood. (2006) 108:3746–52. 10.1182/blood-2006-03-010322 16926295

[26] Petri B Broermann A Li H Khandoga AG Zarbock A Krombach F. von Willebrand factor promotes leukocyte extravasation. Blood. (2010) 116:4712–9. 10.1182/blood-2010-03-276311 20716766

[27] Kanaji S Orje JN Kanaji T Kamikubo Y Morodomi Y Chen Y. Humanized GPIbalpha-von Willebrand factor interaction in the mouse. Blood Adv. (2018) 2:2522–32. 10.1182/bloodadvances.2018023507 30287479 PMC6177644

[28] Chen J Reheman A Gushiken FC Nolasco L Fu X Moake JL. N-acetylcysteine reduces the size and activity of von Willebrand factor in human plasma and mice. J Clin Invest. (2011) 121:593–603. 10.1172/JCI41062 21266777 PMC3026714

[29] Brophy TM Ward SE McGimsey TR Schneppenheim S Drakeford C O'Sullivan JM. Plasmin cleaves von willebrand factor at K1491–R1492 in the A1–A2 linker region in a shear- and glycan-dependent manner *in vitro*. Arterioscler Thromb Vasc Biol. (2017) 37:845–55. 10.1161/ATVBAHA.116.308524 28279966

[30] Sudoyo AW Rachman A Harimurti K. Angiogenesis, inflammation, platelets count, and metastatic status as a predictor for thrombosis risk in nasopharyngeal carcinoma patients. Acta Med Indonesiana. (2015) 47:11–5. 25948762

[31] Ghasemzadeh M Hosseini E. Platelet-leukocyte crosstalk: Linking proinflammatory responses to procoagulant state. Thrombosis Res. (2013) 131:191–7. 10.1016/j.thromres.2012.11.028 23260445

[32] Bollée G Patey N Cazajous G Robert C Goujon JM Fakhouri F. Thrombotic microangiopathy secondary to VEGF pathway inhibition by sunitinib. Nephrol Dialysis Transpl. (2009) 24:682–5. 10.1093/ndt/gfn657 19054798

[33] Erpenbeck L Demers M Zsengellér ZK Gallant M Cifuni SM Stillman IE. ADAMTS13 Endopeptidase protects against vascular endothelial growth factor inhibitor-induced thrombotic microangiopathy. J Am Soc Nephrol. (2016) 27:120–31. 10.1681/ASN.2014121165 26038528 PMC4696577

[34] Habe K Wada H Ito-Habe N Hatada T Matsumoto T Ohishi K. Plasma ADAMTS13, von Willebrand factor (VWF) and VWF propeptide profiles in patients with DIC and related diseases. Thrombosis Res. (2012) 129:598–602. 10.1016/j.thromres.2011.10.011 22070827

[35] Zitomersky NL Demers M Martinod K Gallant M Cifuni SM Biswas A. ADAMTS13 Deficiency worsens colitis and exogenous ADAMTS13 administration decreases colitis severity in mice. TH Open Companion J thrombosis Haemostasis. (2017) 1:e11–e23. 10.1055/s-0037-1603927 29376146 PMC5782810

[36] Zhou S Jiang S Guo J Xu N Wang Q Zhang G. ADAMTS13 protects mice against renal ischemia-reperfusion injury by reducing inflammation and improving endothelial function. Am J Physiol Renal Physiol. (2019) 316:F134–f45. 10.1152/ajprenal.00405.2018 30461292

[37] van Dorland HA Mansouri Taleghani M Sakai K Friedman KD George JN Hrachovinova I. The international hereditary thrombotic thrombocytopenic purpura registry: key findings at enrolment until 2017. Haematologica. (2019). 10.3324/haematol.2019.216796. [Epub ahead of print]. 30792199 PMC6886414

[38] Galbusera M Noris M Remuzzi G. Inherited thrombotic thrombocytopenic purpura. Haematologica. (2009) 94:166–70. 10.3324/haematol.2008.002493 19181790 PMC2635388

[39] Motto DG Chauhan AK Zhu G Homeister J Lamb CB Desch KC. Shigatoxin triggers thrombotic thrombocytopenic purpura in genetically susceptible ADAMTS13–deficient mice. J Clin Invest. (2005) 115:2752–61. 10.1172/JCI26007 16200209 PMC1240119

[40] Banno F Kokame K Okuda T Honda S Miyata S Kato H Complete deficiency in ADAMTS13 is prothrombotic, but it alone is not sufficient to cause thrombotic thrombocytopenic purpura. Blood. (2006) 107:3161–6. 10.1182/blood-2005-07-2765 16368888

[41] Wagstaff L Kelwick R Decock J Edwards DR. The roles of ADAMTS metalloproteinases in tumorigenesis and metastasis. Front Biosci. (2011) 16:1861–72. 10.2741/3827 21196270

[42] Rodríguez-Manzaneque JC Fernández-Rodríguez R Rodríguez-Baena FJ Iruela-Arispe ML. ADAMTS proteases in vascular biology. Matrix Biol. (2015) 44–46:38–45. 10.1016/j.matbio.2015.02.004 25698314 PMC8086761

[43] Rodriguez-Manzaneque JC Lane TF Ortega MA Hynes RO Lawler J Iruela-Arispe ML. Thrombospondin-1 suppresses spontaneous tumor growth and inhibits activation of matrix metalloproteinase-9 and mobilization of vascular endothelial growth factor. Proc Natl Acad Sci USA. (2001) 98:12485–90. 10.1073/pnas.171460498 11606713 PMC60080

[44] Lee M Rodansky ES Smith JK Rodgers GM. ADAMTS13 promotes angiogenesis and modulates VEGF-induced angiogenesis. Microvasc Res. (2012) 84:109–15. 10.1016/j.mvr.2012.05.004 22626948

[45] Lee M Keener J Xiao J Long Zheng X Rodgers GM. ADAMTS13 and its variants promote angiogenesis via upregulation of VEGF and VEGFR2. Cell Mol Life Sci. (2015) 72:349–56. 10.1007/s00018-014-1667-3 24950743 PMC11113207

[46] Bongers TN de Maat MP van Goor ML Bhagwanbali V van Vliet HH Gómez García EB. High von Willebrand factor levels increase the risk of first ischemic stroke: influence of ADAMTS13, inflammation, and genetic variability. Stroke. (2006) 37:2672–7. 10.1161/01.STR.0000244767.39962.f7 16990571

[47] Warlo EMK Pettersen AR Arnesen H Seljeflot I. vWF/ADAMTS13 is associated with on-aspirin residual platelet reactivity and clinical outcome in patients with stable coronary artery disease. Thrombosis J. (2017) 15:28. 10.1186/s12959-017-0151-3 29200971 PMC5700557

[48] Hanson E Jood K Karlsson S Nilsson S Blomstrand C Jern C. Plasma levels of von Willebrand factor in the etiologic subtypes of ischemic stroke. J Thrombosis Haemostasis. (2011) 9:275–81. 10.1111/j.1538-7836.2010.04134.x 21054779

[49] Sonneveld MA Franco OH Ikram MA Hofman A Kavousi M de Maat MP. Von willebrand factor, ADAMTS13, and the risk of mortality: the rotterdam study. Arterioscl Thrombosis Vasc Biol. (2016) 36:2446–51. 10.1161/ATVBAHA.116.308225 27737864

[50] Bongers TN de Bruijne EL Dippel DW de Jong AJ Deckers JW Poldermans D. Lower levels of ADAMTS13 are associated with cardiovascular disease in young patients. Atherosclerosis. (2009) 207:250–4. 10.1016/j.atherosclerosis.2009.04.013 19439298

[51] Denorme F Kraft P Pareyn I Drechsler C Deckmyn H Vanhoorelbeke K. Reduced ADAMTS13 levels in patients with acute and chronic cerebrovascular disease. PLoS ONE. (2017) 12:e0179258. 10.1371/journal.pone.0179258 28591212 PMC5462472

[52] Sonneveld MA de Maat MP Portegies ML Kavousi M Hofman A Turecek PL. Low ADAMTS13 activity is associated with an increased risk of ischemic stroke. Blood. (2015) 126:2739–46. 10.1182/blood-2015-05-643338 26511134

[53] Qu L Jiang M Qiu W Lu S Zhao Y Xia L. Assessment of the diagnostic value of plasma levels, activities, and their ratios of von willebrand factor and ADAMTS13 in patients with cerebral infarction. Clin Appl Thrombosis Hemostasis. (2016) 22:252–9. 10.1177/1076029615583347 25916953

[54] Andersson HM Siegerink B Luken BM Crawley JT Algra A Lane DA. High VWF, low ADAMTS13, and oral contraceptives increase the risk of ischemic stroke and myocardial infarction in young women. Blood. (2012) 119:1555–60. 10.1182/blood-2011-09-380618 22110247

[55] Stoll M Rühle F Witten A Barysenka A Arning A Strauss C Rare variants in the ADAMTS13 von willebrand factor-binding domain contribute to pediatric stroke. circulation. Cardiovasc Genetics. (2016) 9:357–67. 10.1161/CIRCGENETICS.115.001184 27412500

[56] Lambers M Goldenberg NA Kenet G Kirkham FJ Manner D Bernard T. Role of reduced ADAMTS13 in arterial ischemic stroke: a pediatric cohort study. Ann Neurol. (2013) 73:58–64. 10.1002/ana.23735 23225307 PMC3703929

[57] Arning A Hiersche M Witten A Kurlemann G Kurnik K Manner D. A genome-wide association study identifies a gene network of ADAMTS genes in the predisposition to pediatric stroke. Blood. (2012) 120:5231–6. 10.1182/blood-2012-07-442038 22990015

[58] Zhang F Yang XC Jia XW Tang XH Wang Z Wang ZQ. Von Willebrand factor and ADAMTS13 plasma in older patients with high CHA2DS2–VASc Score with and without atrial fibrillation. Eur Rev Med Pharmacol Sci. (2017) 21:4907–12. 29164570

[59] Zhao BQ Chauhan AK Canault M Patten IS Yang JJ Dockal M. von Willebrand factor-cleaving protease ADAMTS13 reduces ischemic brain injury in experimental stroke. Blood. (2009) 114:3329–34. 10.1182/blood-2009-03-213264 19687510 PMC2759655

[60] Fujioka M Hayakawa K Mishima K Kunizawa A Irie K Higuchi S. ADAMTS13 gene deletion aggravates ischemic brain damage: a possible neuroprotective role of ADAMTS13 by ameliorating postischemic hypoperfusion. Blood. (2010) 115:1650–3. 10.1182/blood-2009-06-230110 19965676

[61] Khan MM Motto DG Lentz SR Chauhan AK. ADAMTS13 reduces VWF-mediated acute inflammation following focal cerebral ischemia in mice. J Thrombosis Haemostasis. (2012) 10:1665–71. 10.1111/j.1538-7836.2012.04822.x 22712744 PMC3419774

[62] Dhanesha N Prakash P Doddapattar P Khanna I Pollpeter MJ Nayak MK. Endothelial cell-derived von willebrand factor is the major determinant that mediates von willebrand factor-dependent acute ischemic stroke by promoting postischemic thrombo-inflammation. Arterioscl Thrombosis Vasc Biol. (2016) 36:1829–37. 10.1161/ATVBAHA.116.307660 27444201 PMC5001895

[63] Zhao L Hua C Li Y Sun Q Wu W. miR-525–5p inhibits ADAMTS13 and is correlated with Ischemia/reperfusion injury-induced neuronal cell death. Int J Clin Exp Med. (2015) 8:18115–22. 26770408 PMC4694308

[64] South K Denorme F Salles-Crawley II De Meyer SF Lane DA. Enhanced activity of an ADAMTS-13 variant (R568K/F592Y/R660K/Y661F/Y665F) against platelet agglutination in vitro and in a murine model of acute ischemic stroke. J Thrombosis Haemostasis. (2018) 16:2289–99. 10.1111/jth.14275 30152919 PMC6282751

[65] Yancopoulos GD Klagsbrun M Folkman J. Vasculogenesis, angiogenesis, and growth factors: ephrins enter the fray at the border. Cell. (1998) 93:661–4. 10.1016/S0092-8674(00)81426-9 9630209

[66] Eklund L Saharinen P. Angiopoietin signaling in the vasculature. Exp Cell Res. (2013) 319:1271–80. 10.1016/j.yexcr.2013.03.011 23500414

[67] Starke RD Ferraro F Paschalaki KE Dryden NH McKinnon TA Sutton RE. Endothelial von willebrand factor regulates angiogenesis. Blood. (2011) 117:1071–80. 10.1182/blood-2010-01-264507 21048155 PMC3035068

[68] Saint-Lu N Oortwijn BD Pegon JN Odouard S Christophe OD de Groot PG. Identification of galectin-1 and galectin-3 as novel partners for von Willebrand factor. Arterioscl Thrombosis Vasc Biol. (2012) 32:894–901. 10.1161/ATVBAHA.111.240309 22267483

[69] Markowska AI Jefferies KC Panjwani N. Galectin-3 protein modulates cell surface expression and activation of vascular endothelial growth factor receptor 2 in human endothelial cells. J Biol Chem. (2011) 286:29913–21. 10.1074/jbc.M111.226423 21715322 PMC3191032

[70] Kaikita K Soejima K Matsukawa M Nakagaki T Ogawa H. Reduced von Willebrand factor-cleaving protease (ADAMTS13) activity in acute myocardial infarction. J Thrombosis Haemostasis. (2006) 4:2490–3. 10.1111/j.1538-7836.2006.02161.x 16898955

[71] McCabe DJ Murphy SJ Starke R Harrison P Brown MM Sidhu PS Relationship between ADAMTS13 activity, von Willebrand factor antigen levels and platelet function in the early and late phases after TIA or ischaemic stroke. J Neurol Sci. (2015) 348:35–40. 10.1016/j.jns.2014.10.035 25498844

[72] Yang S Jin M Lin S Cataland S Wu H. ADAMTS13 activity and antigen during therapy and follow-up of patients with idiopathic thrombotic thrombocytopenic purpura: correlation with clinical outcome. Haematologica. (2011) 96:1521–7. 10.3324/haematol.2011.042945 21606162 PMC3186314

[73] Bustamante A Ning M García-Berrocoso T Penalba A Boada C Simats A. Usefulness of ADAMTS13 to predict response to recanalization therapies in acute ischemic stroke. Neurology. (2018) 90:e995–1004. 10.1212/WNL.0000000000005162 29444972 PMC5874450

[74] Fan M Xu H Wang L Luo H Zhu X Cai P. Tissue plasminogen activator neurotoxicity is neutralized by recombinant ADAMTS 13. Sci Rep. (2016) 6:25971. 10.1038/srep25971 27181025 PMC4867598

[75] Denorme F Langhauser F Desender L Vandenbulcke A Rottensteiner H Plaimauer B. ADAMTS13–mediated thrombolysis of t-PA-resistant occlusions in ischemic stroke in mice. Blood. (2016) 127:2337–45. 10.1182/blood-2015-08-662650 26929275

[76] Nakano T Irie K Hayakawa K Sano K Nakamura Y Tanaka M. Delayed treatment with ADAMTS13 ameliorates cerebral ischemic injury without hemorrhagic complication. Brain Res. (2015) 1624:330–5. 10.1016/j.brainres.2015.07.027 26254727

[77] Xu H Cao Y Yang X Cai P Kang L Zhu X. ADAMTS13 controls vascular remodeling by modifying VWF reactivity during stroke recovery. Blood. (2017) 130:11–22. 10.1182/blood-2016-10-747089 28428179 PMC5501147

[78] Prochazka V Jonszta T Czerny D Krajca J Roubec M Macak J. The role of von willebrand factor, ADAMTS13, and cerebral artery thrombus composition in patient outcome following mechanical thrombectomy for acute ischemic stroke. Med Sci Monitor Int Med J Exp Clin Res. (2018) 24:3929–45. 10.12659/MSM.908441 29887594 PMC6029516

